# Investigating a Selection of Methods for the Prediction of Total Soluble Solids Among Wine Grape Quality Characteristics Using Normalized Difference Vegetation Index Data From Proximal and Remote Sensing

**DOI:** 10.3389/fpls.2021.683078

**Published:** 2021-06-11

**Authors:** Aikaterini Kasimati, Borja Espejo-Garcia, Eleanna Vali, Ioannis Malounas, Spyros Fountas

**Affiliations:** Natural Resources Management and Agricultural Engineering, Agricultural University of Athens, Athens, Greece

**Keywords:** normalized difference vegetation index, linear regression, ensemble methods, correlation, quality prediction, wine grape quality, remote sensing, precision viticulture

## Abstract

The most common method for determining wine grape quality characteristics is to perform sample-based laboratory analysis, which can be time-consuming and expensive. In this article, we investigate an alternative approach to predict wine grape quality characteristics by combining machine learning techniques and normalized difference vegetation index (NDVI) data collected at different growth stages with non-destructive methods, such as proximal and remote sensing, that are currently used in precision viticulture (PV). The study involved several sets of high-resolution multispectral data derived from four sources, including two vehicle-mounted crop reflectance sensors, unmanned aerial vehicle (UAV)-acquired data, and Sentinel-2 (S2) archived imagery to estimate grapevine canopy properties at different growth stages. Several data pre-processing techniques were employed, including data quality assessment, data interpolation onto a 100-cell grid (10 × 20 m), and data normalization. By calculating Pearson’s correlation matrix between all variables, initial descriptive statistical analysis was carried out to investigate the relationships between NDVI data from all proximal and remote sensors and the grape quality characteristics in all growth stages. The transformed dataset was then ready and applied to statistical and machine learning algorithms, firstly trained on the data distribution available and then validated and tested, using linear and nonlinear regression models, including ordinary least square (OLS), Theil–Sen, and the Huber regression models and Ensemble Methods based on Decision Trees. Proximal sensors performed better in wine grapes quality parameters prediction in the early season, while remote sensors during later growth stages. The strongest correlations with the sugar content were observed for NDVI data collected with the UAV, Spectrosense+GPS (SS), and the CropCircle (CC), during Berries pea-sized and the Veraison stage, mid-late season with full canopy growth, for both years. UAV and SS data proved to be more accurate in predicting the sugars out of all wine grape quality characteristics, especially during a mid-late season with full canopy growth, in Berries pea-sized and the Veraison growth stages. The best-fitted regressions presented a maximum coefficient of determination (*R*^2^) of 0.61.

## Introduction

Precision viticulture (PV) is a strategy to manage vineyard variability by utilizing spatiotemporal data and observations, to enhance the oenological potential of a vineyard. In addition, new technologies introduced in support of vineyard management allow for the efficiency and quality of production to be improved, and in parallel, minimizing impacts on the environment (Balafoutis et al., 2017). This is particularly relevant in regions, where high wine production quality standards warrant adopting site-specific management practices to increase grape quality and yield.

Grape quality is a complex concept that refers to achieving optimal grape composition characteristics (Dai et al., 2011). Among these, sugar and titratable acidity are commonly used to describe the quality of wine grapes at harvest. The sugar content relates to the wine concentration of alcohol after fermentation, whereas the acid content determines the taste and stability of wine (Herrera et al., 2003). The most common method used in determining wine grape quality characteristics is to perform sample-based laboratory analysis by obtaining the chemical compounds of the grapes, which can be a time-consuming, complex, and expensive process (Cortez et al., 2009).

In recent years, remote sensing is widely applicable in agriculture, specifically crop growth monitoring and crop quality and yield estimation. For example, the normalized difference vegetation index (NDVI) is a vegetation index (VI) used for spatial decision-making in vineyards (Acevedo-Opazo et al., 2008). Canopy response and NDVI can be obtained in a direct, precise, and non-destructive way from various sensors and sensor configurations to acquire different bands, using proximal, aerial, and satellite platforms, based on the distance to the assessed crop (Hall et al., 2011; Baluja et al., 2012). Presently, advanced sensing techniques have had many applications beyond their original scope, especially as computing power has drastically increased in recent years, which have allowed for more complex machine learning techniques to be used to find patterns and correlations between NDVI and specific crop quality and yield characteristics (Pantazi et al., 2016; Kamilaris and Prenafeta-Boldú, 2018).

Previous research has been conducted to estimate crop quality and yield with the assessment of VIs derived from various sensors. A common approach is to perform statistical and regression analysis, including descriptive statistics, Pearson’s correlation, and regression models. The Pearson correlation coefficient has been quantified in various studies to identify the spatial correlation between NDVI and crop quality and yield (Sun et al., 2017; He et al., 2018), research dedicated to selecting key variables to predict the product quality and yield with satisfactory performance directly. Linear and multivariate regression models have been constructed and fitted to various VIs to determine the field-wide production for multiple crops, such as wheat, corn, soybean, sorghum, rice, and grapes (Magney et al., 2016; Sun et al., 2017; Petersen, 2018; Prasetyo et al., 2018). An application on table grapes by Anastasiou et al. (2018) estimated yield and quality with the assessment of VIs derived from satellite and proximal sensing at different growth stages, from veraison to harvest. The VIs exhibited different degrees of correlations with different measurement dates and sensing methods. This study showed that both satellite-based and proximal-based NDVI at both stages (veraison and harvest) presented good correlations to crop quality characteristics, with proximal sensing proving to be the most accurate in estimating table grape yield and quality characteristics. In addition to linear regression models, more advanced approaches for yield estimation have been evaluated using ensemble methods. Boosted Regression Trees, Decision Trees, and Random Forests-based machine learning approaches were used to train the models to estimate crop yield from a short time series of remotely sensed NDVI (Heremans et al., 2015; Bhatnagar and Gohain, 2020).

While previous research has studied various correlation and regression models between VIs and crop production, machine learning techniques for estimating grape quality and yield have not been thoroughly investigated yet. In this article, we propose an alternative approach to predict wine grape quality characteristics by combining machine learning techniques and NDVI data collected at different growth stages with non-destructive methods, such as proximal and remote sensing, that are currently used in PV. For this reason, extensively used regression methods have been compared against more complex methods that deal better with outliers. Finally, to evaluate and ensure the robustness of the machine learning models used in this study, a 5-fold cross-validation procedure was followed across 20 experiments as a validation technique.

## Materials and Methods

### Study Area

The field site where the study was conducted was a commercial wine grape vineyard block on the Palivos Estate located in Nemea, Greece (37.8032°, 22.69412°, WGS84). The vineyard, planted with *Vitis vinifera* L. cv. “Agiorgitiko” for winemaking is located on a steep slope, and the experimental area selected for data collection was approximately 2 ha. Wine grapes were trained to a vertical shoot positioned, cane pruned double Guyot training/trellis system, with northeast-southwest row orientation and row distance of 2.2 m.

### Canopy Reflectance Data Collection

**Figure 1:**
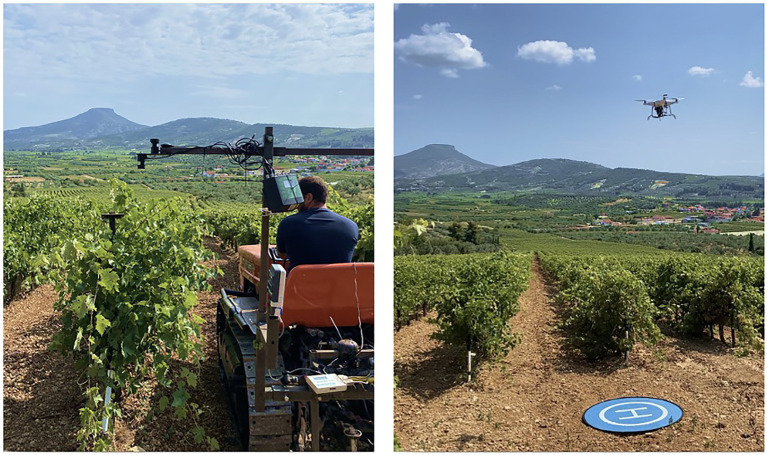
Two vehicle-mounted crop reflectance sensors, UAV-acquired data, and Sentinel-2 archived imagery were used to estimate grapevine canopy properties. UAV, unmanned aerial vehicle.

Canopy reflectance was measured four times per growing season, during 2019 and 2020, starting in late May until the harvest in early September, to record the NDVI at different phenological growth stages of the grapevines. Crop vigor was assessed at these four berry growth stages, namely, (i) Flowering, (ii) Setting, (iii) Berries pea-sized, and (iv) Veraison, with two vehicle-mounted on-the-go proximal sensors, while an unmanned aerial vehicle (UAV) and Sentinel-2 (S2) satellite imagery were used to assess the crop vigor through remote sensing (Figure 1; Table 1). A CropCircle (CC), an active proximal canopy sensor (ACS-470, Holland Scientific Inc., Lincoln, NE, United States), and a Spectrosense+GPS (SS) passive sensor (Skye Instruments Ltd., Llandrindod Wells, United Kingdom) were mounted on a tractor, located at a height of approximately 1.5 m from the soil surface and according to each growth stage, and 0.5 m horizontally from the vines, to record proximal reflectance measurements from the side and the top of the canopy, respectively, at a rate of 1 reading per second and moving at a constant speed of 8–10 km/h. Tractor steering and the relative position of the sensors remained consistent throughout the data collection since the row distance is 2.2 m. All recorded data were georeferenced with a Garmin GPS16X HVS (Garmin, Olathe, Kansas United States) and SS’s built-in GPS. Aerial imagery data were acquired on the same dates as the proximal measurements, with a Phantom 4 Pro UAV (Dà-Jiāng Innovations, Shenzhen, Guangdong, China) equipped with a multispectral Parrot Sequoia+ camera (Parrot SA, Paris, France) and its GPS, enabling to geotag all obtained images. The UAV data acquisition was performed on the same days as the proximal measurements, close to solar noon, with nadir flights at 30 m above ground height. The flight duration was approximately 10 min, and the capture interval of the multispectral camera was set at 2 s. The UAV flight plan overlap and sidelap were 80 and 70%, respectively, with a ground sampling distance (GSD) of the imagery ortho-mosaics ~3 cm. Specifically, atmospherically corrected S2 satellite images, 2A products with a 10 m pixel spatial resolution, were downloaded from the official Copernicus Open Access Hub^1^ for the closest dates available to the dates of the proximal and UAV surveys.

**Table 1:** Grapevine seasonal EL growth stages of proximal and remote sensing data acquisition.

Stage	EL No	Description	Date range	Data acquisition date
Flowering	23	16–20 leaves separated; 50% caps off	June 1–June 20	CC; SS; UAV: 040619, 050620 S2: 130619, 190620
Setting	27	Young berries enlarging, bunch at right angles to stem	June 20–July 20	CC; SS; UAV: 040719, 140720 S2: 050719, 120720
Berries pea-sized	31	About 7 mm in diameter	July 20–Aug 15	CC; SS; UAV: 010819, 110820 S2: 020819, 110820
Veraison	35	Berries begin to color and enlarge	Aug 15–Sept 10	CC; SS; UAV: 280819, 260820 S2: 280819, 280820

CC, CropCircle; SS, Spectrosense+GPS; UAV, unmanned aerial vehicle; and S2, Sentinel-2.

### Data Preparation

**Figure 2:**
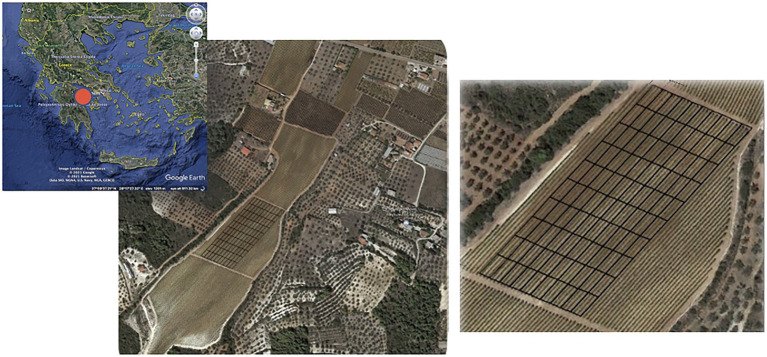
Satellite images of the wine grapes commercial vineyard and the 100-cell grid developed parallel to the trellis lines (Google Earth Pro, 2021).

All proximal canopy reflectance data was transformed to projected coordinates (UTM Zone 34N), cleaned by removing the data points located outside the field boundaries, and interpolated (Taylor et al., 2007). The interpolated data was upscaled to 10 × 20 m cells using ArcMap v10.3 (ESRI, Redlands, CA, United States). This resulted in 100 plots across the study area and generated NDVI maps’ time-series with 10 × 20 m spatial resolution, oriented parallel to the trellis lines (Figure 2). Similarly, the UAV-acquired imagery was combined using Pix4D software (Pix4D S.A., Prilly, Switzerland), and the generated NDVI ortho-mosaic was fitted to the vineyard’s boundaries. Radiometric calibration was applied to the generated ortho-mosaic using the reference images of a radiometric calibration target (Airinov Aircalib), captured after each flight. The software automatically recognized the albedo values for each band. The data was then upscaled using a mean aggregation approach to the same 100 plots. The Sentinel-2 NDVI was calculated using bands 4 and 8, red and near-infrared, respectively, obtaining imagery with 10 m pixel spatial resolution. To place the raster dataset to the spatially correct geographic location, a spatial correction “shift,” based on ground control points from the UAV detailed map was carried out, following the boundaries of the experimental field before the satellite imagery was upscaled to the 10 × 20 m plots by averaging the NDVI of any pixel centroids within the management plots. The last step of satellite image processing was to clip the NDVI according to the border of the experimental field.

### Qualitative Characteristics Analysis

Wine grapes were hand-harvested at the end of each growing season, in mid-September. A regular 100-cell grid (10 × 20 m), covering the total area, was configured to facilitate field sampling to assess crop yield and wine grape quality. The total yield was determined by counting the total number of crates filled with grapes per cell, multiplying it with the average crate weight of the harvested wine grapes. Wine grape quality characteristics were assessed by randomly picking 50 berries from each vineyard cell. The qualitative analysis of substances in berries, musts, and wines, namely, the total soluble solids (°Brix), the total titratable acidity, and the pH were determined.

### Statistical Analysis and Regression Methods

An initial descriptive statistical analysis was executed to assess proximal and remote sensing performance on the prediction of wine grape quality. The exploratory correlation analysis included calculating Pearson’s correlation matrix to investigate the relationships between NDVI data from all four proximal and remote sensors and the wine grape quality characteristics.

Regression model analysis was also performed. Due to various possible distributions found in the input data, several algorithms were evaluated only for those data that presented Pearson’s correlation, with absolute values higher than 0.5 (|*r*| > 0.50). The regression algorithms used were both linear and nonlinear, depending on the output model generated. The linear models used included Ordinary Least Square (OLS), Theil–Sen, and Huber regression models.

- Ordinary least square: It is the most common estimation method for computing linear regression models, which can be found in related works (Prasetyo et al., 2018). The OLS regression is a powerful analysis that can analyze multiple variables simultaneously to answer complex research questions. However, like many statistical analyses, it has several underlying assumptions describing properties of the error term. Moreover, this method can be used only if the data is normally distributed since outliers tend to pull the fitted model far from the accurate result.
- Theil–Sen estimator method: Linear models are sensitive to outliers, and few outliers can skew our predictions heavily (Sen, 1968). Compared with the OLS estimator, the Theil–Sen estimator is robust against outliers. Contrary to OLS, this algorithm uses a generalization of the median instead of the mean. Moreover, it is the most popular non-parametric technique for estimating a linear trend and does not assume the underlying distribution of the input data.
- Huber regression: It is also considered a robust linear regression modeling method, less sensitive to outliers in data (Huber, 1973). Huber regression is aware of the possibility of outliers in a dataset and assigns them less weight than other examples in the dataset, contrary to Theil–Sen that ignores their presence.

Moreover, to improve our model’s predictive power, nonlinear methods, Decision Trees, and different Ensemble methods based on Decision Trees, including AdaBoosting, Random Forests, and Extra Trees were evaluated in the context of this research, combining the predictions from multiple machine learning algorithms together to make more accurate predictions than the individual models. These ensemble methods take one or more decision trees and then reduce their variance and bias by applying them to boost or bootstrap aggregation (bagging).

- Decision trees: Although it can also be used for classification, the algorithm is suitable for regression problems. Decision Tree models are the foundation of all tree-based models, visually representing the “decisions” used to generate predictions. This method uses a non-parametric learning approach. Its main advantage is its straightforward interpretation. If the model is not too complex, it can be visualized to understand better why the classifier made a specific decision. Its major disadvantage is that singular decision tree models are prone to overfitting, resulting in weak, unstable predictions that could have negative consequences if the input data contain noise.
- AdaBoost: The AdaBoost (adaptive boosting) algorithm uses an ensemble-learning approach known as boosting (Freund and Schapire, 1995). First, a decision tree is retrained several times, increasing the emphasis on those data samples where the regression was inaccurate. Then, it combines the predictions from multiple “weak learners,” simple decision tree models, which are added sequentially to the ensemble, correcting the predictions made by the model before it in the sequence.
- Random forest: A supervised learning algorithm that uses ensemble learning method for regression, aggregating many decision tree regressors into one model, which have been trained on different data samples drawn from the input feature (the NDVI in this study), with the bootstrap sampling technique (Breiman, 2004). As a result, the trees in random forests run in parallel, and each tree draws a random sample from the original dataset, adding some randomness that prevents overfitting.
- Extremely randomized trees: Extra Trees are similar to the Random Forest, combining the predictions from many decision trees (Geurts et al., 2006). However, it does not use bootstrap sampling but the entire original input sample. It creates many unpruned decision or regression trees from the training dataset, and predictions are made by averaging the prediction of the decision trees. It uses a random split for node creation to grow the trees, leading to a reduction in overfitting.

Although tree-based methods provide an approach for overcoming the constraints of parametric models, their limitation is that they are computationally more expensive than the traditional OLS. However, if the differences in the performances are high enough, they should be a good approach for addressing the regression modeling problem.

### Fine-Tuning

Optimizing machine learning models relies on an empirical approach, and specifically, in ensemble methods, the number of estimators is not predefined. Moreover, some tree-based hyperparameters, such as the ideal maximum depth of the trees, are unknown until several values are evaluated. Thus, being able to test several model hyperparameters quickly is imperative in maximizing performance. For that reason, to start the training process, several experiments were performed by grid-search to find the best hyperparameters for this study. Table 2 summarizes the primary hyperparameters that governed the ensemble methods. In the results section, the ones that obtained the best performances will be stated.

**Table 2:** Hyperparameters evaluated for optimizing the ensemble learning models.

Hyperparameter	Description	Values
Number of trees	The number of trees used in the ensemble. High values could reduce overfitting.	10, 50, 100, 200
Max. depth	The maximum depth of the trees. Too high could lead to overfitting.	No maximum, 1, 5, 7
Split criteria	The function to measure the quality of a node split. The calculation of the Gini Index is computationally faster, but it could lead to more poor results.	Information gain, Gini impurity

### Evaluation Methodology

The prediction accuracy was assessed using the coefficient of determination (*R*^2^), and root mean square error (RMSE) metrics. Additionally, to test the generalization ability of every regression model and ensure their robustness, a 5-fold cross-validation procedure was followed for each of them. Additionally, to compute the final performances more accurately, the experiments were repeated 20 times with different data splits.

### Software and Hardware

**Figure 3:**
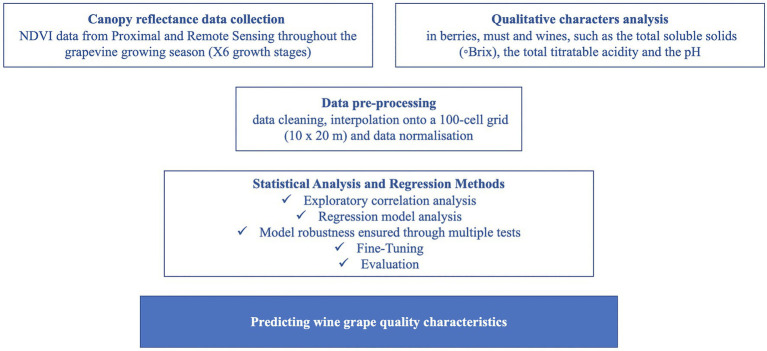
Workflow for investigating a selection of methods for predicting wine grape quality characteristics using normalized difference vegetation index (NDVI) data from proximal and remote sensing.

One main software package was used in this work: Scikit-Learn machine learning library (version 0.23.2). In addition, all the experiments were run on Ubuntu 18.04 as the OS (Figure 3).

## Results

### Exploratory Correlation Analysis

The exploratory correlation analysis included calculating Pearson’s correlation matrix to investigate the relationships between NDVI data from all four proximal and remote sensors and the wine grape quality characteristics. The 2019 and 2020 correlation matrices generally indicated good absolute correlations between NDVI data from all four proximal and remote sensors and total soluble solids, the sugar content measured in °Brix, (|*r*| > 0.50). However, this was not the case for the other two main wine grape quality characteristics, the total titratable acidity, and the pH, that presented no correlation with the NDVI data at any crop stage.

In the top two rows of Table 3, the best intra-sensor correlations between NDVI data and total soluble solids are presented. The signal stabilizes mid-late in the growing season. The maximum correlation (|*r*| = 0.74) for 2019 was observed for Spectrosense+GPS data during Berries pea-sized and the Veraison stage (i.e., mid-late season with full canopy growth). For 2020, correlations were strongest for UAV data (|*r*| = 0.79), at the same growth stages. All Sentinel-2 NDVI variables demonstrated relatively weak correlations (0.29 < |*r*| < 0.57) when correlated with the total soluble solids. For the given stages of the growing season, it was noticed that during Veraison, the NDVI data from UAV, Spectrosense+GPS, and the CC sensors correlated the best with the total soluble solids for both years.

**Table 3:** Selected best performed Pearson’s correlation coefficients comparisons between NDVI data from all four proximal and remote sensors and total soluble solids (i) for a given sensor (rows 1 and 2) and (ii) for given growth stages of the season (rows 3 and 4; CC, CropCircle; SS, Spectrosense+GPS; UAV; and S2, Sentinel-2).

Per sensor	CropCircle	Spectrosense+GPS	UAV	Sentinel-2
2019	0.69 (Veraison) 0.52 (Berries pea-sized)	0.74 (Veraison) 0.69 (Berries pea-sized)	0.63 (Veraison) 0.62 (Flowering)	0.57 (Berries pea-sized) 0.54 (Veraison)
2020	0.54 (Setting) 0.50 (Berries pea-sized)	0.70 (Setting) 0.67 (Veraison)	0.79 (Veraison) 0.77 (Berries pea-sized)	0.33 (Veraison) 0.29 (Setting)
Per growth stage	Flowering	Setting	Berries pea-sized	Veraison
2019	0.62 (UAV) 0.36 (S2)	0.58 (UAV) 0.48 (S2)	0.69 (SS) 0.61 (UAV)	0.74 (SS) 0.69 (CC)
2020	0.76 (UAV) 0.63 (SS)	0.70 (SS) 0.54 (CC)	0.77 (UAV) 0.50 (CC, SS)	0.79 (UAV) 0.67 (SS)

**Figure 4:**
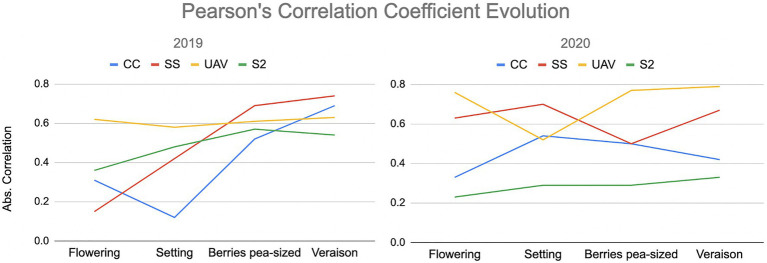
Pearson’s correlation coefficients evolution throughout the growing seasons 2019 and 2020 (legend as for Table 3).

The Pearson’s correlation coefficient evolution for both 2019 and 2020 is shown in Figure 4. The highest correlations between the proximal‐ and remote-based spectral vegetation indices and the wine grape total soluble solids at different crop stages are recorded for the UAV, with the Spectrosense+GPS, the CropCircle, and the Sentinel-2 imagery following. Even though no pattern was noted in the correlation coefficient evolution, it is clear that mid-late season the NDVI correlates the best with wine grape quality characteristics.

### Regression Analysis

Regression model analysis was performed only for those data that presented Pearson’s correlation for NDVI data from all four proximal and remote sensors and total soluble solids, with absolute values higher than 0.5 (|*r*| > 0.50) for the different crop stages. The regression algorithms used, both linear and nonlinear regression analysis, were performed using those highly correlated NDVI data to evaluate their performance in assessing the wine grapes’ quality characteristics. The regression models between NDVI data from all four proximal and remote sensors and total soluble solids presented different degrees of accuracy, depending on the model fitted, the sensor used, and the growth stage assessed. The best fitted, linear, and nonlinear regressions were observed for UAV and Spectrosense+GPS data during the mid-late season with full canopy growth in Berries pea-sized and the Veraison growth stages.

When using OLS, Theil-Sen and Huber linear regression models the best fit for the models was for the estimation of total soluble solids, during Veraison, with a coefficient of determination *R*^2^ ranging from (0.38 < *R*^2^ < 0.61) for both 2019 and 2020. The maximum coefficient of determination for the linear regression models (*R*^2^ = 0.61) was observed for 2020 retrieved with UAV data and fitting the Theil–Sen regression model. For 2019, the OLS seems to perform better using canopy reflectance data coming from the CropCircle and the Spectrosense+GPS proximal sensors. The selected best-performed results of the linear regression analysis are presented in Table 4.

**Table 4:** Selected best performed linear regression models performed using the highly correlated NDVI data from all four proximal and remote sensors to evaluate their performance in assessing the wine grapes quality characteristics (legend as for Table 3).

	Sensor_growth stage	Method	*R*^2^ (avg)	RMSE
2019	SS_Veraison	OLS	0.51 ± 0.09	1.45 ± 0.19
	CC_Veraison	OLS	0.42 ± 0.10	1.67 ± 0.35
	SS_Berries pea-sized	Huber	0.41 ± 0.11	1.71 ± 0.24
	UAV_Veraison	Theil-Sen	0.38 ± 0.10	1.95 ± 0.55
2020	UAV_Veraison	Theil-Sen	0.61 ± 0.03	1.37 ± 0.19
	UAV_Berries pea-sized	Huber	0.57 ± 0.04	1.55 ± 0.32
	UAV_Flowering	Theil-Sen	0.56 ± 0.06	1.75 ± 0.19
	SS_Setting	OLS	0.44 ± 0.07	1.73 ± 0.23
	UAV_Setting	OLS	0.44 ± 0.04	2.09 ± 0.45

Among the nonlinear methods, and to improve our models’ predictive power, different Ensemble methods based on Decision Trees, including AdaBoosting, Random Forests, and Extra Trees, were evaluated, aggregating the predictions from multiple machine learning algorithms together to make more accurate predictions than the individual models. The best fit for the nonlinear model was for estimating total soluble solids, during Veraison, with the coefficient of determination *R*^2^ ranging from (0.42 < *R*^2^ < 0.59) for both 2019 and 2020. The maximum coefficient of determination for the nonlinear regression models (*R*^2^ = 0.59) was observed for 2020 retrieved with UAV data and using the AdaBoost algorithm. For 2019, the Extra Trees performs better using canopy reflectance data from the CropCircle and the Spectrosense+GPS proximal sensors. In the case of the Adaboost, the best hyperparameters were 50 decision trees with a maximum depth of 1. In the case of the Extra Trees, the best hyperparameters were also 50 decision trees, but with a maximum depth of 7 and the split criteria based on Information Gain. This same configuration was the one that led to the highest performance in Random Forest (SS_Berries pea_sized and UAV_Flowering). It is important to note that although the Decision Tree classifier, as a standalone classifier, was also evaluated, its performance was always lower than the ensemble methods. The selected best-performed results of the nonlinear regression analysis are presented in Table 5.

**Table 5:** Selected best performed nonlinear regression models performed using the highly correlated NDVI data from all four proximal and remote sensors to evaluate their performance in assessing the wine grapes quality characteristics (legend as for Table 3).

	Sensor_growth stage	Method	*R*^2^ (avg)	RMSE
2019	SS_Veraison	Extra Trees	0.46 ± 0.03	1.64 ± 0.14
	CC_Veraison	Random Forest	0.43 ± 0.5	1.68 ± 0.32
	SS_Berries pea-sized	Random Forest	0.43 ± 0.06	1.82 ± 0.29
	UAV_Veraison	Extra Trees	0.42 ± 0.05	2.05 ± 0.51
	UAV_Flowering	Random Forest	0.39 ± 0.07	2.11 ± 0.33
2020	UAV_Veraison	AdaBoost	0.59 ± 0.05	1.41 ± 0.25
	UAV_Berries pea-sized	Extra Trees	0.53 ± 0.03	1.65 ± 0.22
	UAV_Flowering	Extra Trees	0.56 ± 0.05	1.72 ± 0.29
	SS_Setting	Extra Trees	0.43 ± 0.02	1.83 ± 0.33
	UAV_Setting	Extra Trees	0.41 ± 0.06	1.95 ± 0.35

## Discussion

In this article, an alternative approach to predict wine grape quality characteristics by combining machine learning techniques and NDVI data collected at different growth stages with non-destructive methods, such as proximal and remote sensing, currently used in precision viticulture, is used in precision viticulture proposed. While previous research has studied various correlation and regression models between VIs and crop production, the use of machine learning techniques for the estimation of grape quality and yield has not been thoroughly investigated yet (Sun et al., 2017; He et al., 2018; Liakos et al., 2018). This study proved that advanced sensing techniques may have many applications, especially with the help of the increasing computing power, allowing for more complex machine learning techniques to find patterns and correlations between canopy reflectance data and specific crop quality characteristics. Furthermore, extensively used regression methods have been compared against more complex methods that deal better with outliers. In addition, to evaluate and ensure the robustness of the machine learning models used in this study, a 5-fold cross-validation procedure was followed across 20 experiments. The validation technique avoids overoptimistic (or random) results based on hold-out evaluations.

Wine grapes quality refers to the achievement of optimal levels of all grape composition characteristics, with sugar content being a basic one, related to the wine concentration of alcohol after fermentation. The exploratory correlation analysis presented that the recorded canopy reflectance data from all four sensors, i.e., the pure vine NDVI extracted from two proximal sensors, a CropCirle and a Spectrosense+GPS and the “mixed pixel” UAV and Sentinel-2 imagery, showed an increasing correlation to the total soluble solids as the season progressed. Similar results have also been found by other researchers (Tagarakis et al., 2013; Fountas et al., 2014), with this study being aligned with Anastasiou et al. (2018), who estimated yield and quality with the assessment of vegetation indices derived from satellite and proximal sensing at different growth stages and their study showed that NDVI at late developmental stages of the vine growing season presented good correlations to crop quality characteristics. Also, García-Estévez et al. (2017) found that the highest correlation of NDVI derived from proximal sensing with yield parameters of wine grapes was at veraison. Sun et al. (2017), the best crop stage for estimating wine grape yield characteristics from satellite-derived data is before harvest. The strongest correlations with the sugar content were observed for NDVI data collected with the UAV, Spectrosense+GPS, and the CropCircle, during Berries pea-sized and the Veraison stage, mid-late season with full canopy growth, for both years. The weaker correlation coefficients recorded with Sentinel-2 and assessed with an overhead “mixed pixel” approach indicated less reliability for wine grapes quality characteristics predictions, which is a sensible result, as Khaliq et al. (2019) also discussed that satellite imagery resolution could not be directly used to describe vineyard variability reliably. However, this was not the case for the other two main wine grape quality characteristics, the total titratable acidity, and the pH, that presented no correlation with the NDVI data at any crop stage.

The performance of each sensor was different and affected by data acquisition parameters, such as proximity to the vines and the specific technical characteristics of the equipment used. The CropCircle and the Spectrosense+GPS proximal sensors were mounted on a tractor, recording reflectance measurements from the side and the top of the canopy, respectively, while the UAV and Sentinel-2 satellite imagery assessed the crop vigor through remote sensing from the top. The highest correlations between the proximal‐ and remote-based spectral vegetation indices and the wine grape total soluble solids at different crop stages are recorded for the UAV, with the Spectrosense+GPS, the CropCircle, and the Sentinel-2 imagery following. The Spectrosense+GPS and UAV seemed to perform better and in a similar way, most probably due to the scanning orientation, which was the top side of the canopy at close proximity. Even though the UAV is classed as a remote sensor, it provides high spatial resolution. Although no pattern was noted in the correlation coefficient evolution, it is clear that the NDVI correlates the best with wine grape quality characteristics in the mid-late season. The exploratory analysis acted as an evaluation for performing predictive analytics on the dataset.

The dataset was then used for training machine learning algorithms, evaluating linear and nonlinear regression models, including OLS, Theil–Sen, and the Huber regression models and Ensemble Methods based on Decision Trees. The regression algorithms used, both linear and nonlinear regression analysis, were performed using those highly correlated NDVI data to evaluate their performance in assessing the wine grapes’ quality characteristics. The regression models between NDVI data from all four proximal and remote sensors and total soluble solids presented different degrees of accuracy, depending on the model fitted, the sensor used, and the growth stage assessed. The UAV and the Spectrosense+GPS data proved to be more accurate in predicting the sugars out of all wine grape quality characteristics, especially during the mid-late season with full canopy growth, in Berries pea-sized and the Veraison growth stages.

All regression methods that were applied, both linear and nonlinear, the OLS, Theil–Sen, and Huber regression models, Decision Trees and Ensemble methods based on Decision Trees, including AdaBoosting, Random Forests, and Extra Trees performed similarly in wine grapes quality parameters prediction, with the best-fitted models achieving a coefficient of determination of *R*^2^ = 0.61. These results confirm the findings of Bhatnagar and Gohain (2020), who used decision tree and random forest-based machine learning approaches to estimate crop yield by comparing their values with NDVI values, and they concluded with the result of *R* = 0.67. Therefore, the implementation of machine learning techniques resulted in similar results as linear models. However, more precise wine grape quality predictions were obtained when NDVI data were collected close to the harvest date, although promising results were obtained for the early season, as noted by Ballesteros et al. (2020). The fact that the ensemble methods performed in some cases slightly worse than the linear methods could be due to the limited dataset size in combination with the use of 5-fold cross-validation, which reduces the training set to 80% of the total dataset size. However, this is considered necessary to provide reliable results. On the other hand, it could be discussed whether stacking learning techniques instead of boosting or bagging could lead to better performances (Wolpert, 1992; van der Laan et al., 2007).

## Conclusion

In this paper, the use of machine learning techniques to estimate wine grape quality characteristics is investigated. An alternative approach investigating the combination of a selection of methods extensively used regression methods to more complex methods that deal better with outliers, predicted wine grape quality characteristics using NDVI data, collected at different growth stages from proximal and remote sensing, is proposed. This study proved that advanced sensing techniques may have many applications, especially with the help of the increasing computing power, allowing for more complex machine learning techniques to find patterns and correlations between canopy reflectance data and specific crop quality characteristics.

The descriptive statistical analysis showed that the NDVI data from the UAV, Spectrosense+GPS, and the CropCircle, during Berries pea-sized and the Veraison stage, mid-late season with full canopy growth, have the strongest correlations with the sugar content for both years. At the same time, Sentinel-2 imagery indicated less reliability for wine grapes’ quality characteristics predictions. The predictive analysis indicated that regression models between NDVI data from all four proximal and remote sensors and total soluble solids presented different degrees of accuracy, depending on the model fitted, the sensor used, and the growth stage assessed. All regression methods that were applied, both linear and nonlinear, performed similarly in wine grapes quality parameters prediction. The UAV and the Spectrosense+GPS data proved to be more accurate in predicting the sugars out of all wine grape quality characteristics, especially closer to the harvesting period. Although correlation is not significant, it seems enough to predict wine grape quality with satisfied approximation.

The investigation of a selection of methods, including OLS, Theil–Sen, and Huber regression models, Decision Trees, AdaBoost, Random Forests, and Extra Trees, for the assessment of wine grape quality characteristics using spectral vegetation indices, presents a great potential for machine learning techniques to be used as an alternative method, to the currently widely used linear regression processes. Ensemble methods presented similar results to regression analysis, while dealing better with the outliers and ensuring robustness through cross-validation techniques. This research will be extended by assessing stacking learning techniques instead of boosting and bagging as a new ensemble method and exploring if they could lead to better performances. Finally, given the perennial nature of grapevines and the various environmental and endogenous factors determining quality, seasonal calibration for quality prediction should be considered in future research.

## Data Availability Statement

The raw data supporting the conclusions of this article will be made available by the authors, without undue reservation.

## Author Contributions

All authors contributed to conception and design of the study. AK organized and realized the data collection and the data pre-processing and preparation, and drafted the manuscript outline. BE-G, EV, and IM contributed to the statistical analysis and machine learning implementation. EV and IM wrote the first draft of the introductory sections of the manuscript. AK, BE-G, and SF completed the results and discussion sections. All authors contributed to the article and approved the submitted version.

### Conflict of Interest

The authors declare that the research was conducted in the absence of any commercial or financial relationships that could be construed as a potential conflict of interest.

## Footnotes

^1^https://scihub.copernicus.eu/
